# Successful management of carinal shift in aortic dissection: A case report of innovative airway management strategies

**DOI:** 10.1002/ccr3.9246

**Published:** 2024-08-03

**Authors:** Rahul Kumar Chaudhary, Sajjad Ahmed Khan

**Affiliations:** ^1^ Department of Anesthesiology and Critical Care Birat Medical College Teaching Hospital Morang Nepal; ^2^ Birat Medical College Teaching Hospital Morang Nepal

**Keywords:** airway management, aortic dissection, cardiovascular emergencies, carinal shift, critical care

## Abstract

The case underscores the necessity of adaptable airway management strategies in aortic dissection surgeries complicated by carinal shift. Successful lung isolation was achieved using a single‐lumen endotracheal tube after failed attempts with traditional methods and bronchial blockers.

## 1 INTRODUCTION

Aortic dissection represents a catastrophic medical emergency characterized by the separation of the layers within the aortic wall, leading to the formation of a false lumen. It is associated with high morbidity and mortality rates, demanding prompt diagnosis, and intervention. Stanford Type B aortic dissection, as classified by the Stanford classification system, involves the descending aorta distal to the left subclavian artery. While medical management serves as the initial approach for uncomplicated cases, surgical intervention becomes imperative in complicated scenarios or when there's a heightened risk of complications such as aortic rupture or malperfusion syndromes.^1^, ^2^

The successful surgical repair of Stanford Type B aortic dissection is often challenged by anatomical distortions within the mediastinum, including carinal shift. Carinal shift denotes the displacement of the trachea and mainstem bronchi due to the expansion of the false lumen or hematoma within the mediastinum. This phenomenon introduces a unique set of challenges for both anesthesiologists and surgeons during airway management and surgical procedures, potentially compromising airway patency and accessibility.^3^, ^4^

Understanding the intricacies of carinal shift and its implications for airway management is crucial for optimizing patient outcomes during the surgical repair of Stanford Type B aortic dissection. Therefore, this case report aims to present a challenging clinical scenario of carinal shift encountered during the surgical repair of Stanford Type B aortic dissection. Additionally, it discusses the strategies employed for successful airway management in this complex setting.

## 2 CASE REPORT

### 2.1 Case history/examination

A 36‐year‐old male patient with a diagnosis of aortic dissection presented at the hospital for surgical repair. On the day of surgery, the patient was induced, but attempts for double‐lumen tube (DLT) insertion were unsuccessful. Fiberoptic bronchoscope (FOB) examination revealed carinal shift towards the right side, and due to multiple intubation attempts, the patient developed vocal cord edema. Ventilation was managed with a single‐lumen endotracheal tube (SLT) until the patient was fully awake. The surgery was postponed, and a new plan for lung isolation was made.

The following week, the case was induced as planned. However, attempts to insert the DLT were again unsuccessful. The only available bronchial blocker (BB) in the market, EZ blocker, was used. Unfortunately, the EZ blocker's bifurcated distal extensions inserted into the right bronchus instead of staying at the carina, leading to compression of the left‐sided main bronchial lumen. Despite multiple attempts, the BB placement was unsuccessful. Ultimately, an SLT of 8 mm ID was inserted endobronchially into the right bronchus with the aid of FOB. One‐lung ventilation (OLV) was successfully achieved for the surgery, which lasted for 13 h. No issues with OLV were encountered throughout the procedure. At the end of surgery, the endotracheal tube (ETT) was withdrawn and positioned 2 cm above the carina.

## 3 METHODS (DIFFERENTIAL DIAGNOSIS, INVESTIGATIONS, AND TREATMENT)

**FIGURE 1:**
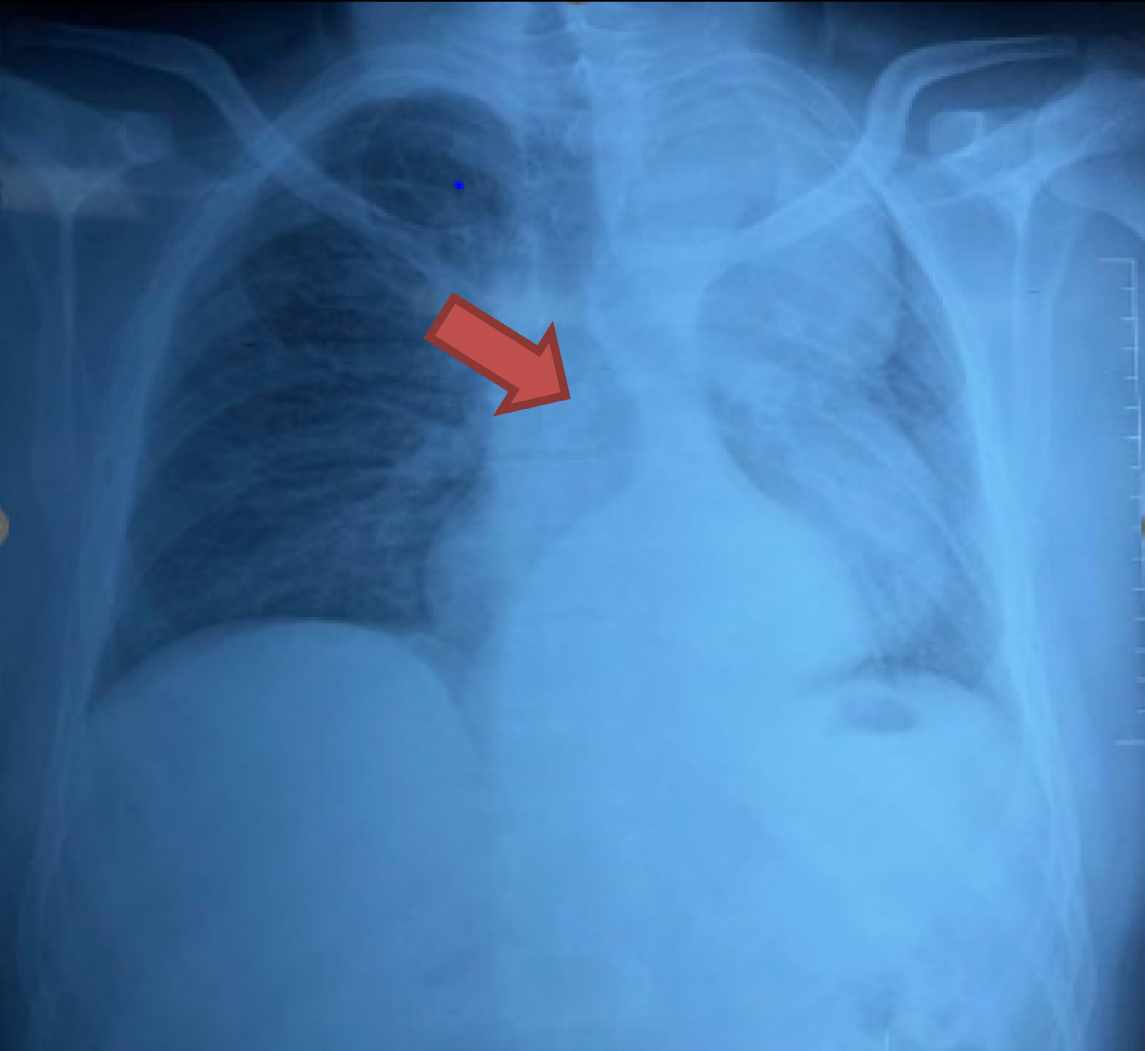
Chest x‐ray PA view showing prominent aortic knuckle and carinal shift.

**FIGURE 2:**
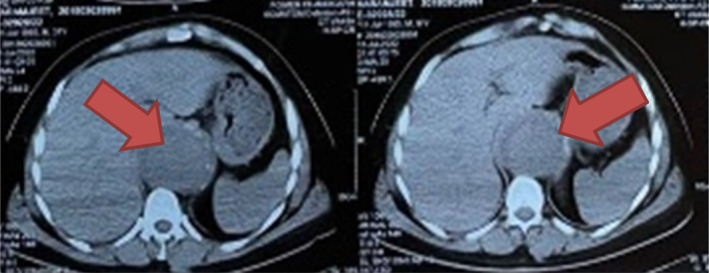
CT Aortogram Axial View showing Stanford Type B Aortic Dissection.

**FIGURE 3:**
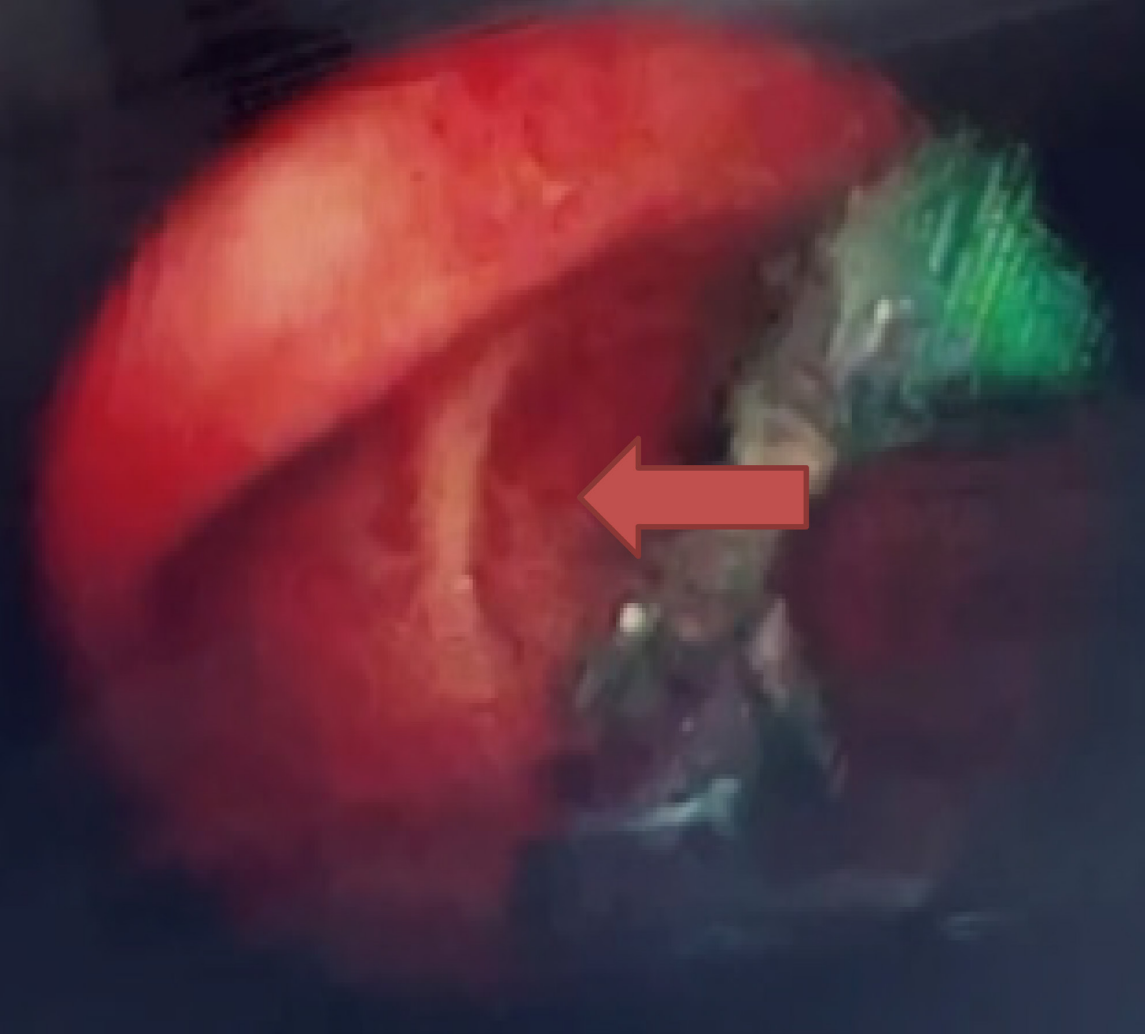
Fiberoptic bronchoscope (FOB) examination showing carinal shift towards the right side.

Pre‐operative investigations, including a chest x‐ray PA view (Figure 1) and CT Aortogram Axial (Figure 2) and coronal views revealed characteristic features indicative of Stanford Type B Aortic Dissection in a 36‐year‐old male patient. These features included a prominent aortic knuckle, widened mediastinum, increased cardiac shadow, expansion of aortic diameter, and double density due to enlargement of the false lumen. Additionally, imaging demonstrated carinal shift, with the dissection commencing at the level of the proximal descending aorta distal to the origin of the left subclavian artery and extending to the aortic bifurcation and bilateral common iliac arteries, with a predominance on the right side. FOB examination further confirmed carinal shift towards the right side, resulting in carinal deviation and displacement of the opening of the left main bronchus away from its usual position due to aortic dissection (Figure 3). Moreover, during FOB examination, the ends of the EZ bronchial blocker were observed to enter the opening of the right main bronchus instead of remaining at the carina, exacerbating the challenges of airway management in this case.

Multiple attempts were made to insert a DLT for lung isolation, but they were unsuccessful. The EZ blocker, the only available BB in the market, was used. However, its bifurcated distal extensions inserted into the right bronchus instead of staying at the carina, causing compression of the left‐sided main bronchial lumen. After failed attempts with the BB, a SLT of 8 mm ID was inserted endobronchially into the right bronchus under fiberoptic guidance. OLV was successfully achieved for the 13‐h surgery using the SLT.

The challenges faced during airway management in this case not only highlight the complexities of anatomical distortion due to Stanford Type B Aortic Dissection but also underscore potential complications associated with the techniques employed. Multiple unsuccessful attempts at DLT insertion can lead to various risks and complications. Each attempt increases the risk of trauma to the airway mucosa, potential for vocal cord injury, and prolongation of anesthesia induction and surgery preparation times. Furthermore, the failure of DLT insertion may necessitate repeated manipulation of the airway, which can exacerbate inflammation and edema, thereby complicating subsequent airway management efforts. The eventual use of a SLT for lung isolation, while effective in achieving OLV in this case, presents its own set of challenges and potential drawbacks. SLTs are not designed specifically for lung isolation and can compromise surgical exposure compared to DLTs. Inadequate isolation may increase the risk of contamination of the non‐operated lung with blood from the dissected aorta, impacting surgical outcomes. Moreover, SLTs may be associated with difficulties in achieving and maintaining OLV, potentially leading to hypoxia or hypercarbia during prolonged surgeries.

Given these considerations, careful assessment of risks and benefits is crucial in selecting the appropriate airway management strategy for patients with complex thoracic anatomy such as severe carinal shift due to aortic dissection. Alternative techniques such as custom‐made bronchial stents or advanced BBs specifically designed for distorted airway anatomy may offer promising solutions in future cases, potentially reducing the risks associated with conventional methods and improving overall patient safety and surgical outcomes.

## 4 CONCLUSION AND RESULT (OUTCOME AND FOLLOW‐UP)

Following the complex surgical repair of Stanford Type B Aortic Dissection with successful lung isolation using a SLT, the postoperative care and monitoring protocols were comprehensive and tailored to ensure optimal recovery and minimize complications.

Initially, the patient was closely monitored in the intensive care unit (ICU) for immediate postoperative management. Continuous monitoring of vital signs, including blood pressure, heart rate, respiratory rate, and oxygen saturation, was essential to detect any signs of hemodynamic instability or respiratory compromise early on. Close observation for any signs of bleeding, fluid imbalance, or neurologic complications related to the surgery or anesthesia was also paramount.

Respiratory care was a priority postoperatively. Given the prolonged surgery and potential for respiratory complications, frequent assessment of lung sounds, respiratory effort, and oxygenation status was conducted. Pulmonary hygiene measures, such as incentive spirometry, deep breathing exercises, and chest physiotherapy, were initiated to promote lung expansion, clear secretions, and prevent atelectasis. Continuous pulse oximetry monitoring ensured timely intervention if oxygenation levels became compromised.

Pain management was another critical aspect of postoperative care. Effective pain control not only enhances patient comfort but also facilitates early mobilization and respiratory function. Multimodal analgesia protocols, including opioids, nonsteroidal anti‐inflammatory drugs (NSAIDs), and local anesthetics, were utilized based on individual patient needs and response. Regular assessment of pain intensity using standardized scales guided adjustments in pain management strategies.

Fluid and electrolyte management were closely monitored to maintain hemodynamic stability and prevent complications such as electrolyte disturbances or fluid overload. Intravenous fluids were administered judiciously based on fluid balance assessments and hemodynamic parameters.

Additionally, postoperative nutritional support was initiated early to optimize healing and recovery. Enteral nutrition via nasogastric tube or oral intake was gradually introduced based on gastrointestinal function and tolerance, ensuring adequate caloric and protein intake to support tissue repair and immune function.

Wound care and monitoring for signs of infection were essential components of postoperative management. Surgical incisions were inspected regularly for signs of healing, drainage, or infection. Prophylactic antibiotics were administered perioperatively according to institutional guidelines to reduce the risk of surgical site infections.

Psychosocial support and patient education were integral to the overall care plan. Clear communication with the patient and family members regarding the surgical procedure, expected recovery trajectory, and potential complications helped alleviate anxiety and fostered collaboration in postoperative care.

The discharge planning process commenced once the patient demonstrated stable vital signs, adequate pain control, and satisfactory progress in mobility and respiratory function. Detailed discharge instructions, including medication reconciliation, follow‐up appointments, and activity restrictions, were provided to facilitate a smooth transition to home care.

Regular follow‐up appointments were scheduled to monitor cardiovascular and respiratory function, assess wound healing, and address any lingering concerns or complications post‐discharge. Multidisciplinary collaboration among cardiologists, pulmonologists, and primary care providers ensured comprehensive long‐term management and ongoing support for the patient's recovery journey.

## 5 DISCUSSION

The presented case highlights the challenges encountered in achieving lung isolation in a patient with aortic dissection complicated by carinal shift. Aortic dissection, particularly Stanford Type B, can lead to anatomical distortions within the mediastinum, including carinal shift, which can complicate airway management during surgical repair.^2^, ^4^

In this case, initial attempts at DLT insertion for lung isolation were unsuccessful due to the patient's carinal shift towards the right side. Carinal shift can result from the expansion of the false lumen or hematoma within the mediastinum, leading to the displacement of the trachea and mainstem bronchi.^5^ The deviation of the trachea and bronchi can make DLT insertion challenging and increase the risk of complications such as vocal cord edema, as seen in this patient.

When traditional methods for lung isolation, such as DLT insertion, failed, alternative strategies were considered. The EZ blocker, a BB with bifurcated distal extensions, was attempted but proved ineffective due to the anatomical distortion caused by the carinal shift. Despite multiple attempts, the EZ blocker's placement led to compression of the left‐sided main bronchial lumen, compromising lung isolation.

Ultimately, a SLT of appropriate size was inserted endobronchially into the right bronchus under fiberoptic guidance. This approach allowed for successful OLV throughout the 13‐h surgical procedure. While SLT insertion for lung isolation is unconventional, it can be a viable alternative in cases where traditional methods fail or are contraindicated.^6^

Successful lung isolation is crucial during aortic dissection surgery to facilitate optimal surgical exposure and prevent contamination of the non‐operated lung with blood from the dissected aorta. Failure to achieve adequate lung isolation can result in suboptimal surgical conditions and increase the risk of intraoperative complications, including impaired ventilation and oxygenation.

Close coordination between the anesthesia and surgical teams is essential in managing challenging airway situations encountered during aortic dissection surgery. Preoperative assessment, including fiberoptic bronchoscopy and imaging studies, can aid in identifying anatomical distortions and planning the appropriate airway management strategy.^7^ Additionally, the availability of alternative airway devices and techniques, such as BBs and SLT insertion, should be considered in cases where traditional methods are unsuccessful.

Recent advancements in managing carinal shift during aortic dissection surgeries have seen progress in both technique and technology. Traditional methods such as DLT insertion may prove challenging in cases of severe carinal shift due to anatomical distortion. Advanced imaging modalities like computed tomography angiography (CTA) play a crucial role in preoperative planning by providing detailed insights into mediastinal anatomy and the extent of carinal deviation.^8^

Innovative approaches include the development of custom‐made endobronchial stents tailored to fit specific anatomical variations encountered in aortic dissection patients.^9^ These stents can be deployed to stabilize the airway and facilitate controlled lung ventilation during surgery, potentially overcoming challenges posed by carinal displacement. Moreover, novel BBs with improved designs are being explored for their efficacy in cases where traditional methods are inadequate. These blockers are designed to navigate complex airway configurations and provide selective lung isolation with minimal risk of complications such as mucosal trauma or obstruction.^10^ This case aligns with established protocols by underscoring the necessity for a multidisciplinary approach involving anesthesia, surgery, and radiology teams to devise tailored strategies for optimal airway management in challenging surgical scenarios. It emphasizes the importance of adapting to individual patient anatomy and employing advanced techniques when traditional approaches fall short.^11^

In conclusion, while recent advancements in managing carinal shift during aortic dissection surgeries show promising results, ongoing research and clinical experience will continue to refine these approaches, aiming to improve outcomes and patient safety in complex thoracic surgical interventions.

## 6 CONCLUSIONS

In conclusion, the presented case underscores the importance of flexibility and adaptability in airway management strategies during surgical interventions for aortic dissection, particularly when confronted with anatomical distortions such as carinal shift. Despite encountering challenges with traditional methods like DLT insertion and BB placement, successful lung isolation was ultimately achieved using a SLT inserted endobronchially under fiberoptic guidance. This case highlights the critical role of multidisciplinary collaboration, meticulous planning, and utilization of alternative techniques in ensuring optimal outcomes for patients undergoing complex surgeries. The successful surgical repair of the aortic dissection without respiratory complications demonstrates the effectiveness of the devised lung isolation strategy. Regular follow‐up and monitoring are essential to ensure the patient's continued recovery and well‐being postoperatively.

## AUTHOR CONTRIBUTIONS

**Rahul Kumar Chaudhary:** Conceptualization; writing – review and editing. **Sajjad Ahmed Khan:** Conceptualization; writing – original draft.

## FUNDING INFORMATION

None.

## CONFLICT OF INTEREST STATEMENT

The authors have no conflict of interest to declare.

## CONSENT

Written informed consent was obtained from the patient to publish this report in accordance with the journal's patient consent policy.

## Data Availability

Datasets generated or analyzed during this study are available from the corresponding author on reasonable request.
